# From Goal Programming for Continuous Multi-Criteria Optimization to the Target Decision Rule for Mixed Uncertain Problems

**DOI:** 10.3390/e24010051

**Published:** 2021-12-28

**Authors:** Helena Gaspars-Wieloch

**Affiliations:** Department of Operations Research and Mathematical Economics, Poznan University of Economics and Business, Al. Niepodleglosci 10, 61-875 Poznań, Poland; Helena.gaspars@ue.poznan.pl

**Keywords:** goal programming, continuous multi-criteria optimization under certainty, neutral criteria, mixed strategies, one-criterion uncertain problems, attitude towards risk, scenario planning, game theory

## Abstract

Goal programming (GP) is applied to the discrete and continuous version of multi-criteria optimization. Recently, some essential analogies between multi-criteria decision making under certainty (M-DMC) and scenario-based one-criterion decision making under uncertainty (1-DMU) have been revealed in the literature. The aforementioned similarities allow the adjustment of GP to an entirely new domain. The aim of the paper is to create a new decision rule for mixed uncertain problems on the basis of the GP methodology. The procedure can be used by pessimists, optimists and moderate decision makers. It is designed for one-shot decisions. One of the significant advantages of the novel approach is related to the possibility to analyze neutral criteria, which are not directly taken into account in existing classical procedures developed for 1-DMU.

## 1. Introduction

Goal programming (GP) is one of the procedures applied to multi-criteria decision making under certainty (M-DMC). This issue is related to the situation where the decision maker (DM) assesses particular courses of action (options, alternatives, decision variants) with the use of more than one criterion and all the parameters of the decision problem are known.

GP was first applied by Charnes et al. [1]. In subsequent years, this procedure has been extended to fuzzy multi-criteria problems [2,3] or combined with other methods for various applications [4,5].

Multi-criteria optimization involves two areas: Multiple Objective Decision Problems (MODP) and Multiple Attribute Decision Problems (MADP). Within MODP, the decision maker formulates and solves a mathematical optimization model with a set of objective functions and a set of constraints on the basis of which the set of possible solutions can be created. However, the number of options is not exactly known [6,7]. In MADP, the number of potential variants is precisely determined at the beginning of the decision making process. Additionally, the levels of analyzed attributes are assigned to each alternative [8]. GP may be used in the continuous and discrete versions of M-DMC, i.e., in MODP and MADP, respectively [9].

It is worth stressing that GP is especially designed for problems where neutral criteria are taken into consideration. “Neutral criteria are neither maximized nor minimized because they consist in reaching a specific value” [10]. The use of neutral criteria is quite frequent in solving real decision problems. They may concern, for instance, “the period of paying off the credit (the term of the loan), the rental time of office space, the duration of the project, the temperature level, the distance between two places, the number of rooms in a house, the surface of the plot or the level of precipitation” [10].

Recently, some vital analogies between multi-criteria decision making under certainty and scenario-based one-criterion decision making under uncertainty (1-DMU) have been revealed and discussed in [9,11]. These similarities allow for the adjustment of goal programming, initially designed for multi-criteria optimization, to an entirely new domain (1-DMU). In [10] the discrete case has been already investigated, which means that a new approach for 1-DMU pure strategy searching has been proposed by referring to the methodology applied within GP for MADP. Nevertheless, the aforementioned analogy in the continuous case has not been explored yet. That is why the aim of this paper is to create a novel method for mixed one-criterion uncertain problems on the basis of the GP ideas developed for MODP. We analyze diverse types of decision makers (pessimist, optimist, moderate). The significant advantage of the new decision rule is related to the possibility of analyzing neutral criteria, which are not directly taken into account in existing classical procedures developed for 1-DMU and mixed strategies [10], i.e., the Bayes, Hurwicz, Wald and max-max optimization models.

The rest of the paper is organized as follows. Section 2 (Materials and Methods) compares pure and mixed strategies; describes the idea of goal programming; reminds its subsequent steps for the continuous version of multi-criteria optimization; and presents M-DMC, 1-DMU and the analogies between both issues. The last part of this section is devoted to the description of a novel approach for 1-DMU and mixed strategies. The suggested procedure is based on the GP (initially designed for multi-criteria decision making). Section 3 (Calculation and Results) contains an illustrative example to show how the new decision rule may be applied to uncertain problems and mixed strategy searching. The characteristics of the suggested method are discussed in Section 4 (Discussion and Conclusions).

## 2. Materials and Methods

### 2.1. Pure and Mixed Strategies

In the previous section, pure and mixed strategies have been mentioned. What do both notions mean? In the case of a pure strategy the decision maker chooses and performs only one decision variant from a set of potential options [9]. For instance, one house from a set of five possible houses is bought, or one project from a set of ten projects is selected.

Nevertheless, in many cases mixed strategies may be more effective [12]. A mixed strategy occurs when the DM chooses and executes a combination of alternatives. Such an approach may be especially useful and advantageous in portfolio construction [13,14] and cultivation of different plants [9]. In the next subsections, the emphasis will be put on mixed strategies.

### 2.2. Goal Programming for MODP

The continuous version of goal programming may be used in different forms, e.g., weighted goal programming, lexicographic goal programming and Chebyshew goal programming [15], but in this paper we focus on the first variety. The steps are as follows:

1. Define the decision variables: *x*_1_, …, *x_j_*, …, *x_n_*.
2. Define the objectives *C*_1_, …, *C_k_*, …., *C_p_* and corresponding objective functions: *f*_1_(*x*), …, *f_k_*(*x*), …, *f_p_*(*x*) where *p* is the number of criteria (the problem may be presented by means of Table 1).
3. Declare the importance of each objective in the form of criteria weights: *w*_1_(*x*), …, *w_k_*(*x*), …, *w_p_*(*x*).
4. Define the desired levels of each criterion *u*_1_, …, *u_k_*, …, *u_p_*.
5. Formulate the synthetic objective function. If criteria are expressed in the same units and scales, use Equation (1). Otherwise, apply Equation (2).
  (1) $$GP\left(x\right)=\sum_{k=1}^{p}w_{k}\left|f_{k}\left(x\right)-u_{k}\right|$$
  (2) $$GP\left(x\right)=\sum_{k=1}^{p}w_{k}\left|g_{k}\left(x\right)-r_{k}\right|$$
  where *g_k_*(*x*) denotes the normalized objective function for criterion *k*, and *r_k_* is the normalized desired level of this criterion. We can assume that the normalization is based on the following formulas (Equations (3) and (5) are related to maximized criteria, Equations (4) and (6) concern minimized criteria):
  (3) $$g_{k}\left(x\right)=\frac{f_{k}\left(x\right)-f_{k}^{min}\left(x\right)}{f_{k}^{max}\left(x\right)-f_{k}^{min}\left(x\right)}$$
  (4) $$g_{k}\left(x\right)=\frac{f_{k}^{max}\left(x\right)-f_{k}\left(x\right)}{f_{k}^{max}\left(x\right)-f_{k}^{min}\left(x\right)}$$
  (5) $$r_{k}=\frac{u_{k}-f_{k}^{min}\left(x\right)}{f_{k}^{max}\left(x\right)-f_{k}^{min}\left(x\right)}$$
  (6) $$r_{k}=\frac{f_{k}^{max}\left(x\right)-u_{k}}{f_{k}^{max}\left(x\right)-f_{k}^{min}\left(x\right)}$$
6. Add constraints describing the decision situation:
  (7) x∈SFS
  where *SFS* denotes the set of feasible solutions.
7. Solve the problem.

The aforementioned optimization model is not easy to solve since the synthetic objective function contains elements with the absolute value. That is why it is recommended to transform the initial problem into a linear optimization model, in connection with the fact that *y_k_* and *z_k_* may represent the following expressions:

(8) $$y_{k}=max\left\{f_{k}\left(x\right)-u_{k};0\right\}\;\mathrm{or}\;y_{k}=max\left\{g_{k}\left(x\right)-r_{k};0\right\}$$

(9) $$z_{k}=max\left\{-f_{k}\left(x\right)+u_{k};0\right\}\;\mathrm{or}\;z_{k}=max\left\{-g_{k}\left(x\right)+r_{k};0\right\}$$

and that:

(10) max{a;0}+max{−a;0}=|a| 

(11) max{a;0}−max{−a;0}=a 

the model (1), (7) or (2), (7) may be simplified to the linear model (12), (7), (13), (15) or (12), (7), (14), (15).

(12) $$GP\left(x,y,z\right)=\overset{p}{\underset{k=1}{\sum }}w_{k}\left(y_{k}+z_{k}\right)\to min$$

(13) $$f_{k}\left(x\right)-y_{k}+z_{k}=u_{k}\;k=1,\ldots ,p$$

(14) $$g_{k}\left(x\right)-y_{k}+z_{k}=r_{k}\;k=1,\ldots ,p\;$$

(15) $$y_{k,}z_{k}\geq 0\;k=1,\ldots ,p\;$$

The above transformation results from the fact that since the absolute value of parameter *a* may be represented by means of Equation (10), similarly the absolute value of the expression $f_{k}\left(x\right)-u_{k}$ may be simplified to $y_{k}+z_{k}$ if we assume that $y_{k}$ and $z_{k}$ denote the expressions given by Equations (8) and (9).

As we see, the essence of the weighted goal programming is to minimize the distances between the realized values of particular criteria and their desired levels. Additionally, the DM has the possibility to declare the importance of a given criterion, so the distances concerning the most significant objectives are the most punished.

The optimal solution obtained after solving the aforementioned models represents a mixed strategy, but note that sometimes the solution generated by the continuous version of the weighted GP may be a simple pure strategy. Such a situation occurs when only one decision variable is positive (and equal to one) and the remaining ones are equal to zero.

When analyzing the construction of the algorithm, we may formulate the following question—why does step 5 take into account maximized and minimized criteria if the essence of GP is to concentrate on neutral criteria, which have a defined desired level? Indeed, in GP neutral criteria are mainly applied, but in order to normalize different values it is conventionally assumed that particular objectives tend to be maximized or minimized.

As a matter of fact, there is another optimization method that may lead to similar (but not identical!) solutions. It is the SAW method (simple additive weighting method), where the sum of weighted normalized values are maximized, but in that case the normalization for neutral criteria requires the use of a different formula.

In this paper, we focus on the case where targets are given as points, but it is worth stressing that the GP may be applied to situations where targets are defined as intervals [10].

### 2.3. Analogies between M-DMC and 1-DMU

Analogies between multi-criteria decision making under certainty and scenario-based one-criterion decision making under uncertainty have been thoroughly discussed in [9,10]. Therefore, in this article we only mention some relationships between issues.

On the one hand, M-DMC is related to cases where the DM assesses particular courses of action in terms of many criteria (at least two) and the parameters of the problem are supposed to be known. On the other hand, 1-DMU “is connected with situations in which the DM evaluates a given decision variant in terms of one objective function, but, due to numerous unknown future factors, the parameters of the problem are not deterministic” [10]. This time a set of potential scenarios is available. These scenarios may be defined by experts, decision makers or by a person who is simultaneously an expert and a DM. “Scenario means a possible way in which the future might unfold” [9]. Scenario planning is a comfortable and relatively simple tool enabling uncertainty modeling [16,17]. There are diverse uncertainty levels [9,18]: I. Uncertainty with known probabilities—the DM knows the options, scenarios, scenario probabilities and particular payoffs; II. Uncertainty with partially known probabilities—the DM knows the options, scenarios, partial scenario probabilities and particular payoffs—probabilities may be given as interval values, sometimes scenarios are ordered according to their approximate chance of occurrence; III. Uncertainty with unknown probabilities—the DM knows the options, scenarios and particular payoffs—scenario probabilities are not known; and IV. Uncertainty with unknown scenarios—the DM knows the options only. In the paper we investigate the third level. The words “payoff”, “result” and “outcome” signify the effect gained by the decision maker if they select a given alternative and a given scenario occurs.

If we compare the structure of the table representing M-DMC (Table 1) with the structure of the table representing 1-DMU (Table 2), we will see a clear similarity.

In both cases, “there is a set of potential options and the set of significant objectives in M-DMC can correspond to the set of possible scenarios in 1-DMU” [9]. Another analogy is related to the final step of the decision making process. The decision maker, in both decision problems, can select and execute only one option (pure strategy) or a combination of several options (mixed strategy).

Of course, the analyzed issues also have essential differences:

1. Within 1-DMU, “if *A_j_* is chosen, the final outcome (*a_i_*_,*j*_) is single and depends on the real scenario which will occur, meanwhile within M-DMC, if *A_j_* is selected, there are *p* final outcomes, i.e., *b*_1,*j*_, …, *b_k_*_,*j*,_ …, *b_p_*_,*j*_, as particular options are evaluated in terms of *p* objectives” [10].
2. In the case of M-DMC “initial values usually have to be normalized since they represent the performance of different criteria which are expressed by means of different scales and units. For 1-DMU the problem concerns one criterion. Thus, the normalization is useless” [9].

Despite the observed differences, relationships between both areas give the opportunity to adjust the initial GP model to a totally new issue, i.e., the scenario-based one-criterion decision making under uncertainty.

### 2.4. New Approach for 1-DMU and Mixed Strategies

By analogy to the weighted goal programming developed for the continuous version of M-DMC, the steps for the new approach could be as follows:

1. Define the decision variables: *x*_1_, …, *x_j_*, …, *x_n_.*
2. Define the scenarios: *S*_1_, …, *S_i_*, …, *S_m_* and corresponding scenario functions: *f*_1_(*x*), …, *f_i_*(*x*), …, *f_m_*(*x*) (the problem may be presented by means of Table 2).
3. Declare the subjective chance of occurrence of particular scenarios: *p*_1_, …, *p_i_*, …, *p_m_*.
4. Define the desired levels of the analyzed criterion within particular scenarios: *u*_1_, …, *u_i_*, …, *u_m_*.
5. Formulate the synthetic objective function.
  (16) $$TDR\left(x\right)=\overset{m}{\underset{i=1}{\sum }}p_{i}\left|f_{i}\left(x\right)-u_{i}\right|\to min$$
6. Add constraints describing the decision situation:
  (17) x∈SFS
7. Solve the problem.

In connection with the fact that the synthetic objective function contains elements with the absolute value, the initial problem can also be transformed in this case to a linear optimization model. It suffices to assume that:

(18) $$y_{i}=max\left\{f_{i}\left(x\right)-u_{i};0\right\}$$

(19) $$z_{i}=max\left\{-f_{i}\left(x\right)+u_{i};0\right\}$$

and to solve the model (20), (17), (21), (22):

(20) $$TDR\left(x,y,z\right)=\overset{m}{\underset{i=1}{\sum }}w_{i}\left(y_{i}+z_{i}\right)\to min$$

(21) $$f_{i}\left(x\right)-y_{i}+z_{i}=u_{i}\;i=1,\ldots ,m\;$$

(22) $$y_{i,}z_{i}\geq 0\;i=1,\ldots ,m\;$$

Step 3 is related to the subjective chance of occurrence. As we mentioned in Section 2.3, the paper focuses on the third level of uncertainty within which the objective probabilities are not known. Nevertheless, when these parameters are unknown, the decision maker may declare the subjective probabilities that represent their attitude towards risk, predictions, state of mind and soul. Within scenario planning, the set of scenarios does not need to be exhaustive, so the sum of subjective probabilities does not need to be equal to one. Note that step 3 from the algorithm for 1-DMU is much more complicated than step 3 within the original interactive programming designed for M-DMC. Decision makers are usually able to declare the importance of particular criteria, but they are not so confident when they are supposed to define the chance of occurrence of a given scenario. Such information does not concern the present, but the future is less known than the present. Therefore, decision makers may want to declare any subjective probabilities, without any additional analysis. On the one hand, a quick probability estimation may speed up the use of the algorithm, but on the other hand, a rapid declaration of these parameters is really not recommended since their level may significantly affect the final solution (see Section 3). Of course, after solving the problem, a sensitivity analysis connected with the subjective chance of occurrence may be performed if the DM intends to check how the solution changes under the influence of probability changes.

Step 4 may also be surprising, since it allows for declaring different desired levels for each scenario, though in each case this level is defined for the same and only criterion considered in the decision problem. The use of varied parameters can be justified in the following way. Sometimes the desired payoff may depend on the scenario; here are two examples. The first one is related to the portfolio construction, where the objective represents the revenue. Normally this criterion should be maximized, but in many countries tax law discourages people and institutions from making too much profit, because exceeding the threshold means a higher tax rate. If within particular scenarios different tax thresholds and tax rates are assumed, the decision maker may be interested in declaring different desired revenue levels for each scenario. The second example concerns a competition, where for each task the player may receive a certain number of points. This number depends on the scenario that will occur. Usually people tend to maximize the number of points. However, if the final prize depends on the number of points obtained and the player is interested in winning a specific award, which is not necessarily connected with the greater number of points, they may decide to treat this criterion as neutral. If, additionally, a given award is not intended for a specific number points, but for a specific place in the ranking, the use of different desired levels may be recommended. So, as we could observe, real decision situations are often more complex than simple theoretical problems. The decision maker has to take into consideration numerous circumstances and factors, which may bring them to the conclusion that the analyzed criterion, usually regarded as maximized or minimized, is neutral from their point of view.

Equation (16) minimizes the weighted deviations from the desired levels. The deviations related to scenarios with the highest subjective chance of occurrence are the most punished.

We have certainly noticed that the GP algorithm for 1-DMU is much less complicated than the original procedure developed for M-DMC, since this time the normalization is not required. It is a significant advantage of the suggested approach.

We have mentioned in the introduction that the novel decision rule would be suitable both for extreme decision makers (optimists and pessimists) and moderate people. It is a vital feature, since many classical decision rules are designed for a limited group of decision makers (see the Wald rule, Savage rule, max-max rule, Hayashi rule, Bayes rule [19]). The use of subjective probabilities enables the adjustment of the model to the decision maker’s attitude towards risk, but sometimes the estimation of these parameters may be quite complex. If for each option the payoffs of a given scenario are smaller than payoffs connected with another scenario, the decision maker has no difficulty in determining the worse scenario and the better one. However, if it is difficult to assign a status to each scenario due to similar payoff ranges, the intuitive estimation of subjective probabilities can be impossible. Therefore, in such cases we recommend the use of an algorithm which facilitates the scenario assessment, e.g., the first stage of the SF + AS procedure described in [20].

## 3. Calculation and Results

Let us call the new decision rule the Target Decision Rule (TDR) for mixed uncertain problems (the abbreviation TDR has been already used in Equation (16)) and analyze a concrete example solved by means of this approach. The investor intends to buy stocks of various companies: *A*_1_, *A*_2_, *A*_3_, *A*_4_ and *A*_5_. The decision variable *x_j_* denotes the share (in percent) of the capital invested in a given company (step 1). They consider six possible scenarios, which differ from each other by the scope of the political policy and the stock market situation. Table 3 represents predicted annual revenues (in EUR) (step 2). We assume that the investor is a *moderate pessimist*. In their opinion, the subjective chance of occurrence of particular scenarios is as follows: 0.15, 0.10, 0.05, 0.10, 0.40 and 0.20 (step 3). The desired levels are equal to 180,000, 180,000, 160,000, 160,000, 90,000 and 90,000, respectively (step 4).

Within step 5, the synthetic objective function is formulated:

(23) $$TDR\left(x\right)=0.15\left|400x_{1}+\ldots +2000x_{5}-180,000\right|+\ldots +0.2\left|600x_{1}+\ldots +350x_{5}-90,000\right|\to min$$

In step 6, the investor may declare some constraints, for instance:

(24) $$0\leq x_{1},\ldots ,x_{5}\leq 35$$

(25) $$x_{1}+\ldots +x_{5}=100$$

Now we can solve the problem, but, in order to simplify the objective function, we are going to transform the model (23)–(25) into (24)–(33):

(26) $$TDR\left(x\right)=0.15\left(y_{1}+z_{1}\right)+\ldots +0.2\left(y_{6}+z_{6}\right)\to min$$

(27) $$y_{1,}z_{1},\ldots ,y_{6,}z_{6}\geq 0$$

(28) $$400x_{1}+\ldots +2000x_{5}-y_{1}+z_{1}=180,000$$

(29) $$1100x_{1}+\ldots +3800x_{5}-y_{2}+z_{2}=180,000$$

(30) $$1200x_{1}+\ldots +3800x_{5}-y_{3}+z_{3}=160,000$$

(31) $$900x_{1}+\ldots +1350x_{5}-y_{4}+z_{4}=160,000$$

(32) $$500x_{1}+\ldots +300x_{5}-y_{5}+z_{5}=90,000$$

(33) $$600x_{1}+\ldots +350x_{5}-y_{6}+z_{6}=90,000$$

where:

(34) $$y_{1}=max\left\{400x_{1}+\ldots +2000x_{5}-180,000;0\right\}$$

(35) $$z_{1}=max\left\{-400x_{1}-\ldots -2000x_{5}+180,000;0\right\}$$

(36) $$y_{2}=max\left\{1100x_{1}+\ldots +3800x_{5}-180,000;0\right\}$$

(37) $$z_{2}=max\left\{-1100x_{1}-\ldots -3800x_{5}+180,000;0\right\}$$

(38) $$y_{3}=max\left\{1200x_{1}+\ldots +1650x_{5}-160,000;0\right\}$$

(39) $$z_{3}=max\left\{-1200x_{1}-\ldots -1650x_{5}+160,000;0\right\}$$

(40) $$y_{4}=max\left\{900x_{1}+\ldots +1350x_{5}-160,000;0\right\}$$

(41) $$z_{4}=max\left\{-900x_{1}-\ldots -1350x_{5}+160,000;0\right\}$$

(42) $$y_{5}=max\left\{500x_{1}+\ldots +300x_{5}-90,000;0\right\}$$

(43) $$z_{5}=max\left\{-500x_{1}-\ldots -300x_{5}+90,000;0\right\}$$

(44) $$y_{6}=max\left\{600x_{1}+\ldots +350x_{5}-90,000;0\right\}$$

(45) $$z_{6}=max\left\{-600x_{1}-\ldots -350x_{5}+90,000;0\right\}$$

The linear model contains 17 non-negative decision variables. The optimal solution is as follows: *x*_1_ = 0.53%, *x*_2_ = 35%, *x*_3_ = 16.10%, *x*_4_ = 35% and *x*_5_ = 13.36%. It means that the investor should allocate 0.53% of their capital to shares A_1_, 35% to shares A_2_, etc. The weighted sum of deviations is equal to 23,554.28 and scenario functions are equal to *f*_1_(*x*) = 89,909.23, *f*_2_(*x*) = 180,000, *f*_3_(*x*) = 201,551.6, *f*_4_(*x*) = 160,000, *f*_5_(*x*) = 72,674.96 and *f*_6_(*x*) = 84,834.66. Thus, for scenarios S_2_ and S_4_, the investor’s revenue would be exactly equal to the desired levels (*y*_2_ = *z*_2_ = *y*_4_ = *z*_4_ = 0). For scenarios S_1_, S_5_ and S_6_ (*z*_1_, *z*_5_, *z*_6_ > 0), this revenue would be lower than the desired levels, and for scenario S_3_ the revenue would be higher (*y*_3_ > 0).

Of course, constraint (24) has a strong impact on the final structure of the portfolio. If we remove this condition, the shares are equal to *x*_1_ = 0%, *x*_2_ = 54.39%, *x*_3_ = 22.97%, *x*_4_ = 0% and *x*_5_ = 22.64%.

Indeed, in the analyzed illustrative example the investor is a moderate pessimist, since the highest subjective chance of occurrence was connected with the scenario with the smallest average of payoffs. If the investor was a *moderate* DM declaring the following values: 0.35, 0.10, 0.05, 0.35, 0.05 and 0.10, the optimal structure of the mixed strategy recommended by TDR would be *x*_1_ = 0%, *x*_2_ = 20.88%, *x*_3_ = 9.13%, *x*_4_ = 35% and *x*_5_ = 35%. The weighted sum of deviations would be equal to 31,629 and scenario functions would equal *f*_1_(*x*) = 121,368.8, *f*_2_(*x*) = 235,956.3, *f*_3_(*x*) = 199,475, *f*_4_(*x*) = 160,000, *f*_5_(*x*) = 59,401.3 and *f*_6_(*x*) = 69,912.5. Thus, for scenario S_4_ the investor’s revenue would be exactly equal to the desired levels (*y*_4_ = *z*_4_ = 0). For scenarios S_1_, S_5_ and S_6_ (*z*_1_, *z*_5_, *z*_6_ > 0), this revenue would be lower than the desired levels, and for scenarios S_2_ and S_3_ the revenue would be higher (*y*_2_,*y*_3_ > 0).

Of course, again, Equation (24) strongly affects the final structure of the portfolio. If we remove this condition, the shares are equal to *x*_1_ = 0%, *x*_2_ = 0%, *x*_3_ = 18.52%, *x*_4_ = 0% and *x*_5_ = 81.48%.

Let us also examine the case of a moderate optimist (the subjective chance of occurrence is equal to 0.10, 0.30, 0.35, 0.15, 0.05 and 0.05, respectively. Then, the optimal solution is: *x*_1_ = 18.47%, *x*_2_ = 35%, *x*_3_ = 0%, *x*_4_ = 31.12% and *x*_5_ = 15.41%, and the weighted sum of deviations equals 15,861.74. Scenario functions are equal to *f*_1_(*x*) = 86,826, *f*_2_(*x*) = 180,000, *f*_3_(*x*) = 160,000, *f*_4_(*x*) = 127,276.3, *f*_5_(*x*) = 67,530 and *f*_6_(*x*) = 79,754.2. Thus, for scenarios S_2_ and S_3_, the investor’s revenue would be exactly equal to the desired levels (*y*_2_ = *z*_2_ *= y*_3_ = *z*_3_ = 0), and for scenarios S_1_, S_4_, S_5_ and S_6_ (*z*_1_, *z*_4_, *z*_5_, *z*_6_ > 0) this revenue would be lower than the desired levels.

In the last situation, Equation (24) also has a decisive influence on the final solution. If we remove this condition, the shares are equal to *x*_1_ = 0%, *x*_2_ = 51.22%, *x*_3_ = 0%, *x*_4_ = 35.17% and *x*_5_ = 13.61%.

Hence, as it can be observed, TDR can be applied by diverse decision makers.

Each problem has been solved by means of SAS/OR (Statistical Analysis System for Operations Research), but the construction of the problem (thanks to the elimination of the absolute values) is so simple that the use of such software as Solver in Excel is also possible.

The fictitious data given in Table 3 may look too unrealistic, but actually the choice of values in this research was rather random. When preparing the example, the only aim was to create scenarios with a high average of payoffs (1970—S_3_, 1850—S_2_), with a moderate average (1550—S_4_) and with a low average (634—S_5,_ 738—S_6_), since such a situation may justify the use of varied subjective chances of occurrence and different desired levels.

In Section 2, we have stressed that the level of subjective probabilities may affect the final solution. Let us assume that the moderate pessimist, instead of values 0.15, 0.10, 0.05, 0.10, 0.40 and 0.20, declares the following probabilities: 0.20, 0.08, 0.02, 0.10, 0.35 and 0.25. They are very similar, because they still assign the highest chance of occurrence (0.35) to the scenario with the worst average of payoffs (S_5_)_,_ the next chance (0.25) to the scenario with a higher average of outcomes (S_6_), etc. If we keep constraint (24), the solution will not change, i.e., the shares will be still equal to *x*_1_ = 0.53%, *x*_2_ = 35%, *x*_3_ = 16.10%, *x*_4_ = 35% and *x*_5_ = 13.36% (of course, the objective function value will change from 23,554.28 to 26,204.28, since it depends on probabilities, but this modification does not affect the investor’s strategy). However, if we analyze the problem with new probability values and without the constraint concerning the maximum possible share (24), the solution will change significantly from *x*_1_ = 0%, *x*_2_ = 54.39%, *x*_3_ = 22.97%, *x*_4_ = 0% and *x*_5_ = 22.64% to *x*_1_ = 0%, *x*_2_ = 39.91%, *x*_3_ = 37.74%, *x*_4_ = 0% and *x*_5_ = 22.35%. This short analysis shows that the DM should declare the subjective chances of occurrence very carefully, especially in the case where decision variables are not bounded because even if the probabilities change only their levels (not their order), the final solution may change.

## 4. Discussion and Conclusions

The aim of this paper was to extend the applications of goal programming originally designed for multi-criteria optimization under certainty. It turns out that thanks to some analogies between M-DMC and scenario-based one-criterion decision making under uncertainty, the ideas of GP may also be applied to the second area, but, of course, the interpretation of the final results is different. In the first case, deviations represent the distances between the desired levels of particular criteria and the real performance of these objectives, while in the second case the deviations show the distances between the desired levels concerning only one criterion and the expected performance of this objective [10]. It is worth emphasizing that TDR (Target Decision Rule, for mixed strategies) is designed for one-shot decisions, i.e., for decisions chosen and executed only once, since after the implementation of the selected strategy, the decision maker has new experiences on the basis of which they can update their attitude towards risk.

The novel approach has three essential advantages:

1. It does not require the normalization of initial values, which means that TDR is less time consuming than the original goal programming designed for M-DMC.
2. It can be used by DMs representing different attitudes towards risk, since one of the steps of the algorithm allows defining the subjective chance of occurrence of subsequent scenarios.
3. It can be applied not only to maximized and minimized criteria, but also to neutral criteria that occur in numerous domains.

TDR for pure strategies described in [10] has the same benefits. The relationship between both procedures is very strong, since if an additional constraint with binary decision variables was introduced to the optimization model used in TDF for mixed strategies, then the final solution would always represent a pure strategy.

Now, let us answer the question “why is the use of the novel approach (TDR for mixed strategies) more advantageous than existing decision rules?”. Popular classical mixed decision rules developed within game theory are: the Wald optimization model, the Bayes optimization model, the max-max optimization model and the Hurwicz optimization model. The Wald rule is only applicable to an extreme pessimist. The Bayes rule refers to repetitive executions, since the average of payoffs is taken into considerations. Thus, it does not fit one-shot decisions. Furthermore, the attitude towards risk cannot be taken into account within this procedure. The max-max approach is merely useful for extreme optimists. The only classical method that could replace TDR for mixed strategies is the Hurwicz optimization model, since this technique enables applying diverse optimism coefficients. Nevertheless, due to the use of the weighted average of payoffs in the objective function, there is no possibility to compare the scenario function values with the desired levels. Additionally, the Hurwicz indices are computed on the basis of the extreme payoffs only. They do not take intermediate values into account, which may lead to illogical recommendations especially in the case of asymmetric payoffs [21]. Originally, classical mixed decision rules were not designed for neutral criteria, but a way to extend their applications could be the use of utility functions that allow for transforming initial payoffs into results, representing the subjective value from the point of view of a given decision maker. In connection with all the observations described above, we can conclude that the strengths of TDR for mixed strategies (compared with existing procedures) are: (1) the possibility to control all the payoffs (not only the selected ones) connected with particular alternatives; (2) the opportunity to generate different solutions (not one solution) for a given payoff matrix, depending on the DM’s predictions; and (3) the possibility to include any type of criterion. The suggested approach fills the research gap identified in the paper.

It is worth noting that, instead of referring to goal programming ideas in the case of a neutral criterion considered in 1-DMU, the optimization model could contain two constraints with an upper and a lower bound for each scenario, but such a way of including neutral objectives would often lead to the formulation of models with contradictory conditions and empty sets of feasible solutions. That is why the use of the goal programming ideas in 1-DMU is so beneficial—in this case, even if the DM declares desired levels difficult to obtain, the model has a solution because the aim of the model is to minimize the distance (between the desired levels and the real ones), not to reach a strictly defined result for each scenario.

Note that the essence of TDR (as with other classical decision rules) is to take the decision maker’s preferences (needs, expectations) into account the best way possible. Parameters used in the optimization model are supposed to reflect their attitude towards risk. Thus, within uncertain decision rules, the emphasis is not put on the final real effect, but on the way the model considers the DM’s state of mind and soul. In connection with this fact, the comparative analysis between the model solution and the actual result is not conducted in this article. Additionally, it is worth underlining that the payoff matrix has a significant impact on the solution generated by the decision rule. Hence, if the expert estimations used in the payoff matrix are entirely wrong, there is little chance of finding an effective strategy, even if the DM tries to apply the approach best suited to their preferences.

When analyzing the results obtained in the previous section, we can notice that TDR for mixed strategies also has a limitation. Though the weights in the synthetic objective function indicate the subjective chance of occurrence, the correlation between this factor and the absolute value of the deviation for each scenario is not always negative and strong. Furthermore, this limitation also occurs in the original version of goal programming for M-DMC—the correlation between criteria weights and the absolute values of the deviation can even be positive when solving a given problem. This means that in the case of GP, when two criteria have the same weight, their deviations from the desired levels may be different in the optimal solution. In addition, in the case of TDR for mixed strategies, if the DM assigns an identical subjective chance of occurrence to two scenarios, their deviations from the desired levels may also be different. This defect has not been revealed in the literature yet, but the observed phenomenon can be treated as a reason to improve the original version of goal programming for M-DMC as well as its new extension, i.e., TDR for mixed strategies. Currently, the lack of strong and negative correlation occurs even when constraints concerning the strategy structure are removed. As a matter of fact, the only situation, where the deviation is indeed always equal to zero for the scenario with the highest subjective probability (provided that there is only one such scenario), occurs when the DM is an extreme optimist or an extreme pessimist (i.e., when only one scenario has a positive subjective chance of occurrence). The same relationship is visible within M-DMC: when there is only one significant criterion (i.e., with a positive weight) and the remaining criteria obtain zero weights, the deviation concerning the aforementioned objective is always equal to zero. Of course, the aforementioned zero deviations in both optimization issues are possible only if the desired levels are properly defined by the DM (they are neither too high nor too low).

Another issue that could be explored in the future is related to the nature of the target. In this article, it was assumed that the target was given as a point, but in real situations this parameter is sometimes given as an interval (e.g., the temperature). Therefore, the model presented in the paper could be extended in the future. 

Note that other possible future research directions are not limited to the issues already described above. The next step could be connected with the creation of a scenario-based hybrid referring to TDR and designed for uncertain multi-criteria problems (M-DMU). Such problems [22,23,24] occur more frequently in real economic decision situations than deterministic multi-criteria problems (M-DMC) or indeterministic one-criterion problems (1-DMU). M-DMU procedures already exist, but they instead focus on pure strategy searching. The mixed strategy searching process on the basis of multiple neutral criteria certainly needs further investigation.

## Figures and Tables

**Table 1:** Payoff matrix for M-DMC.

	Alternatives ^1^				
Criteria	*A*_1_	…	*A_j_*	…	*A_n_*
***C*_1_**	*b*_1,1_	…	*b*_1,*j*_	…	*b*_1,*n*_
⋮	⋮	⋱	⋮	⋱	⋮
***C_k_***	*b_k,_*_1_	…	*b_k,j_*	…	*b_k,n_*
⋮	⋮	⋱	⋮	⋱	⋮
***C_p_***	*b_p,_*_1_	…	*b_p,j_*	…	*b_p,n_*

^1^*n*—number of alternatives, *p*—number of criteria, *b_k,j_*—performance of criterion *C_k_* if option *A_j_* is selected. Source: [9].

**Table 2:** Payoff matrix for 1-DMU with unknown probabilities.

	Alternatives ^2^				
Scenarios	*A*_1_	…	*A_j_*	…	*A_n_*
***S*_1_**	*a*_1,1_	…	*a*_1,*j*_	…	*a*_1,*n*_
⋮	⋮	⋱	⋮	⋱	⋮
***S_i_***	*a_i_*_,1_	…	*a_i,j_*	…	*a_i,n_*
⋮	⋮	⋱	⋮	⋱	⋮
***S_m_***	*a_m,_*_1_	…	*a_m,j_*	…	*a_m,n_*

^2^*n*—number of alternatives, *m*—number of scenarios, *a_i,j_*—payoff obtained if option *A_j_* is selected and scenario *S_i_* occurs. Source: [9].

**Table 3:** Payoff matrix (example).

	Stocks				
Scenarios	*A*_1_	*A*_2_	*A*_3_	*A*_4_	*A*_5_
***S*_1_**	400	500	650	1000	2000
***S*_2_**	1100	1200	1250	1900	3800
***S*_3_**	1200	900	3500	2600	1650
***S*_4_**	900	700	2700	2100	1350
***S*_5_**	500	1000	770	600	300
***S*_6_**	600	1150	850	740	350

Source: prepared by the author.

## Data Availability

All the data used in the paper are fictitious and prepared by the author. Data are given in Table 3. Computations have been made in SAS/OR: https://v4e049.vfe.sas.com/SASStudioV/ (accessed on 6 September 2021)

## References

[1] Charnes A., Cooper W.W., Ferguson R.O. (1955). Optimal estimation of executive compensation by linear programming. Manag. Sci..

[2] Ghaffar A., Razzaq A., Hasan M., Ashraf Z., Khan M.F. (2020). Fuzzy goal programming with an imprecise intuitionistic fuzzy preference relations. Symmetry.

[3] Khan M.F., Hasan M., Quddoos A., Fügenschuh A., Hasan S.S. (2020). Goal programming models with linear and exponential fuzzy preference relations. Symmetry.

[4] Giokas D. (1997). The use of goal programming and data envelopment analysis for estimating efficient marginal costs of outputs. J. Oper. Res. Soc..

[5] Lin H., Nagalingam S., Lin G. (2009). An interactive meta-goal programming-based decision analysis methodology to support collaborative manufacturing. Robot. Comput. Integr. Manuf..

[6] Ding T., Liang L., Yang M., Wu H. (2016). Multiple Attribute Decision Making based on cross-evaluation with uncertain decision parameters. Math. Probl. Eng..

[7] Tzeng G.-H., Huang J.J. (1981). Multiple Attribute Decision Making, Methods and Applications. Lecture Notes in Economics and Mathematical Systems 186.

[8] Singh A., Gupta A., Mehra A. (2020). Matrix games with 2-tuple linguistic information. Ann. Oper. Res..

[9] Gaspars-Wieloch H. (2021). On some analogies between one-criterion decision making under uncertainty and multi-criteria decision making under certainty. Econ. Bus. Rev..

[10] Gaspars-Wieloch H. (2020). A new application for the Goal Programming–the Target Decision Rule for Uncertain Problems. J. Risk Financ. Manag..

[11] Gaspars-Wieloch H. From the interactive programming to a new decision rule for uncertain one-criterion problems. Proceedings of the 16th International Symposium on Operational Research.

[12] Liuzzi G., Locatelli M., Piccialli V., Rass S. (2021). Computing mixed strategies equilibria in presence of switching costs by the solution of nonconvex QP problems. Comput. Optim. Appl..

[13] Latoszek M., Ślepaczuk R. (2020). Does the inclusion of exposure of volatility into diversified portfolio improve the investment results? Portfolio construction from the perspective of a Polish investor. Econ. Bus. Rev..

[14] Zhi B., Wang X., Xu F. (2021). Portfolio optimization for inventory financing: Copula-based approaches. Comput. Oper. Res..

[15] Gür S., Tamer E. (2018). Scheduling and planning in service systems with goal programming: Literature review. Mathematics.

[16] Durbach I.N. (2019). Scenario planning in the analytic hierarchy process. Futures Foresight Sci..

[17] Durbach I.N., Stewart T.J. (2012). Modeling uncertainty in multi-criteria decision analysis. Eur. J. Oper. Res..

[18] Waters D. (2011). Supply Chain Risk Management. Vulnerability and Resilience in Logistics.

[19] Gaspars-Wieloch H. (2020). Critical analysis of classical scenario-based decision rules for pure strategy searching. Organ. Manag. Ser..

[20] Gaspars-Wieloch H. (2015). On a decision rule supported by a forecasting stage based on the decision maker’s coefficient of optimism. Cent. Eur. J. Oper. Res..

[21] Gaspars-Wieloch H. (2014). Modifications of the Hurwicz’s decision rule. Cent. Eur. J. Oper. Res..

[22] Helber S., de Kok T., Kuhn H., Manitz M., Matta A., Stolletz R. (2019). Quantitative approaches in production management. OR Spectr..

[23] Kloos K., Pibernik R., Schulte B. (2019). Allocation planning in sales hierarchies with stochastic demand service-level targets. OR Spectr..

[24] Zhang S., Tang F., Li X., Liu J., Zhang B. (2021). A hybrid multi-objective approach for real-time flexible production scheduling and rescheduling under dynamic environment in Industry 4.0 context. Comput. Oper. Res..

